# New insights in the reproducibility of visual and electronic tooth color assessment for dental practice

**DOI:** 10.1186/s13005-020-00248-w

**Published:** 2020-12-16

**Authors:** Anja Ratzmann, Alexander Welk, Stephanie Hoppe, Jochen Fanghaenel, Christian Schwahn

**Affiliations:** 1 Department of Orthodontics and Department of Dental Propaedeutics/Community Dentistry, Dental School, University Medicine, Walther-Rathenau-Strasse 42, 17475 Greifswald, Germany; 2 Department of Restorative Dentistry, Periodontology, Endodontology, Preventive and Paediatric Dentistry, Dental School, University Medicine, Walther-Rathenau-Strasse 42, 17475 Greifswald, Germany; 3 Private Dental Office, Promenadestrasse, 296047 Bamberg, Germany; 4 Department of Orthodontics, Dental School, University Medicine, Franz-Josef-Strauß-Allee 11, 93053 Regensburg, Germany; 5 Department of Prosthetic Dentistry, Gerontology and Biomaterials, University Medicine, Walther-Rathenau-Strasse 42, 17475 Greifswald, Germany

## Abstract

**Background:**

The aim of the study was to compare a 2D and 3D color system concerning a variety of statistical and graphical methods to assess validity and reliability of color measurements, and provide guidance on when to use which system and how to interpret color distance measures, including ΔE and d(0M1).

**Methods:**

The color of teeth 14 to 24 of 35 patients undergoing regular bleaching treatment was visually assessed and electronically measured with the spectrophotometer Shade Inspector™. Tooth color was recorded before bleaching treatment, after 14 days, and again after 6 months. VITAPAN® Classical (2D) and VITA-3D-Master® (3D) served as reference systems.

**Results:**

Concerning repeated measurements*,* the 2D system was superior to the 3D system, both visually and electronically in terms of ΔE and d(OM1), for statistics of agreement and reliability. All four methods showed strong patterns in Bland-Altman plots. In the 3D system, *hue* was less reliable than *lightness* and *chroma*, which was more pronounced visually than electronically. The smallest detectable color difference varied among the four methods used, and was most favorable in the electronic 2D system. Comparing the methods, the agreement between the 2D and 3D system in terms of ΔE was not *good*. The reliability of the visual and electronic method was essentially the same in the 2D and 3D systems; this comparability is *fair* to *good.*

**Clinical relevance:**

The 3D system may confuse human raters and even electronic devices. The 2D system is the simple and best choice.

## Background

Valid and reliable measurements of tooth color are of major importance in esthetic and restorative dentistry as well as in dental technical practice. Tooth color is usually described based on the Munsell color space in terms of hue, value, and chroma [1, 2]. Hue measures the basic color, value indicates the lightness of a color, and chroma measures the saturation or intensity of a color. Value is determined first, followed by chroma, yielding hue as the third dimension. One of the most important prerequisites is the assessment of tooth color either via visual comparison with prefabricated color scales or using measuring devices such as a colorimeter, spectrophotometer or digital imaging systems with corresponding software [3]. The most common method in clinical practice is still the visual method using *VITAPAN*® *Classical* shade guide, which is a 2D system. In 1998, the *VITA 3D-Master*® shade guide was launched on the dental market. It was developed to systematize color determination, thereby enhancing the likelihood of valid and reliable color measurements [4–7]. Concerning the systematic determination, however, an implicit prior belief about the *VITA 3D Master*® was not checked in developing this color guide: namely, that any two 3D shades within the same dimension at given constant shade values of the other two dimensions can be well differentiated by the human eye. In fact, dentists and dental technicians believe that the third dimension (*hue*) is problematic and that the distance between adjacent 3D shades is not large enough in this dimension. To quantify color differences, ΔE as the Euclidean distance between two points in the color space of the three dimensions (value, chroma, and hue) has been used in the majority of dental color studies [8–20], although a modification of ΔE is preferable [21]. However, numerous studies comparing visual and electronic methods have been published over the past decade [3, 8, 11, 18–20, 22–27].

Taking tooth color measurements is a complex process. In psychology and statistics, it is well known that repeated measurements [28, 29] or groups of observations such as on patients’ teeth increase reliability [30, 31]. Moreover, the favored ΔE to measure color differences cannot be applied to important graphical and statistical methods for the assessment of validity and reliability, including Bland-Altman plots to examine patterns of disagreement and the intraclass correlation coefficient (ICC) to estimate measurement variability [32]. These limitations can be overcome by using the distance of each shade from 0M1 of the 3D color system, denoted by d(0M1) [33]. Because d(0M1) does not distinguish shades of the same radius from M1, d(0M1) and ΔE are complementary rather than competing. For example, in studying bleaching effects, d(OM1) may be favorable for 0M1 but less favorable for comparing shades by gender and age groups (or to study whether the gender difference in tooth color increases with age). In general, validity depends on the purpose [34] and is to be redefined for every research question; there is no such thing as a universal gold standard [35, 36]. Likewise, choosing methods to assess reproducibility depends on the purpose [37]. Whereas reliability is often related to calibration or comparability of examiners before and during performance of large cross-sectional or multicenter studies (only one measurement per participant in the full-scale investigation), the smallest detectable difference or the smallest detectable change is sought in longitudinal studies (at least two measurements per participant; measurement error occured twice or more) [37], when the difference between repeated measurements is in the focus of interest. The smallest detectable difference or, in the present context, the smallest detectable color difference (SDCD), describes a statistical property and is different from perceptible or acceptable color difference thresholds. The SDCD of a row of teeth can easily be recalculated from the SDCD of a single tooth [31]. The SDCD may differ from method to method and from study to study; it contradicts the idea that color difference thresholds are universally valid. In other words, the concept of a universal color difference threshold is scientifically misleading because it confuses validity and reliability. Moreover, color metrics are arbitrary, color perception is subjective, and acceptable color shade differences vary among different colors (ΔE: 1.1 among red shades and 2.1 among yellow shades) [38]. Despite these limitations of color science, it can serve as a rough guide for color difference thresholds and may be useful in daily tooth color determination in dentistry. Therefore, different aspects must be considered when comparing the conventional 2D system with the newer 3D system. This seems more reasonable, because it is more ordered. Ordering alone, however, may not be enough, because the human or electronic rater must have the chance to measure reliably. Whereas directly adjacent shades of the 3D system have mean ΔE values of about 3.8 for *lightness* (1M1-2M1-3M1) and 4.4 for *chroma* (2M1–2M2–2M3), the mean ΔE value is only about 1.5 for the six direct neighbors of *hue* (2L1.5–2R1.5;2L2.5–2R2.5) [38].

Thus, it can be hypothesized that *hue* is measured less reliably than *lightness* or *chroma*. This can be examined not only for an electronic rater but also for a human rater; within-subject comparisons are justified because the examiner serves as her/his own control (*hue* as exposure versus *lightness* or *chroma* as reference), similar to n-of-1 trials [39].

The aim of this study was to compare the 2D and the 3D color systems concerning a variety of statistical and graphical methods to assess validity and reliability, as well as to provide guidance on when to use which system and how to interpret ΔE and d0M1.

## Materials and methods

### Subjects and clinical procedure

In order to better assess clinically relevant color changes, color measurements were performed in patients receiving a regular in-office bleaching treatment (BT). The tooth-inclusion criteria for performing BT were no caries, endodontic treatment or restorations. Patients with insufficient oral hygiene, previous BT, periodontal disease, pregnancy, and allergy or hypersensitivity to the bleaching agents were excluded. The study was approved by the ethics committee of the Medical Association (Ärtzekammer) of Mecklenburg-Vorpommern (Reg. Nr.III UV 15/08). All patients gave informed consent. Thirty-five patients (24 women, 11 men, average age 30 years) from the Dental Clinic at the University of Greifswald participated. The complete clinical procedure was performed under standardized conditions according to the standardized clinical protocol for in-office bleaching under the supervision of an experienced dentist (AW). The bleaching procedure was performed on teeth 15 to 25 and 35 to 45. Supra- and subgingival plaque, stains and calculus were removed, and all teeth were polished with non-fluoridated, oil-free pumice before bleaching.

The gingiva was protected by a liquid gingiva protectant (Dental Dam, Schütz Dental, Rosbach, Germany) activated by a light-curing unit (Ortholux TM LED Lurnig Light, Fa. 3 M Unitek). Bleach’n Smile, 35% H_2_O_2,_ (Schütz Dental, Rosbach, Germany) was applied three times for 10 minutes according to the manufacturer’s recommendation.”

After bleaching, all teeth were fluoridated with Elmex® gelée (CP GABA, Germany).

### Visual and electronic color assessment

**Fig. 1:**
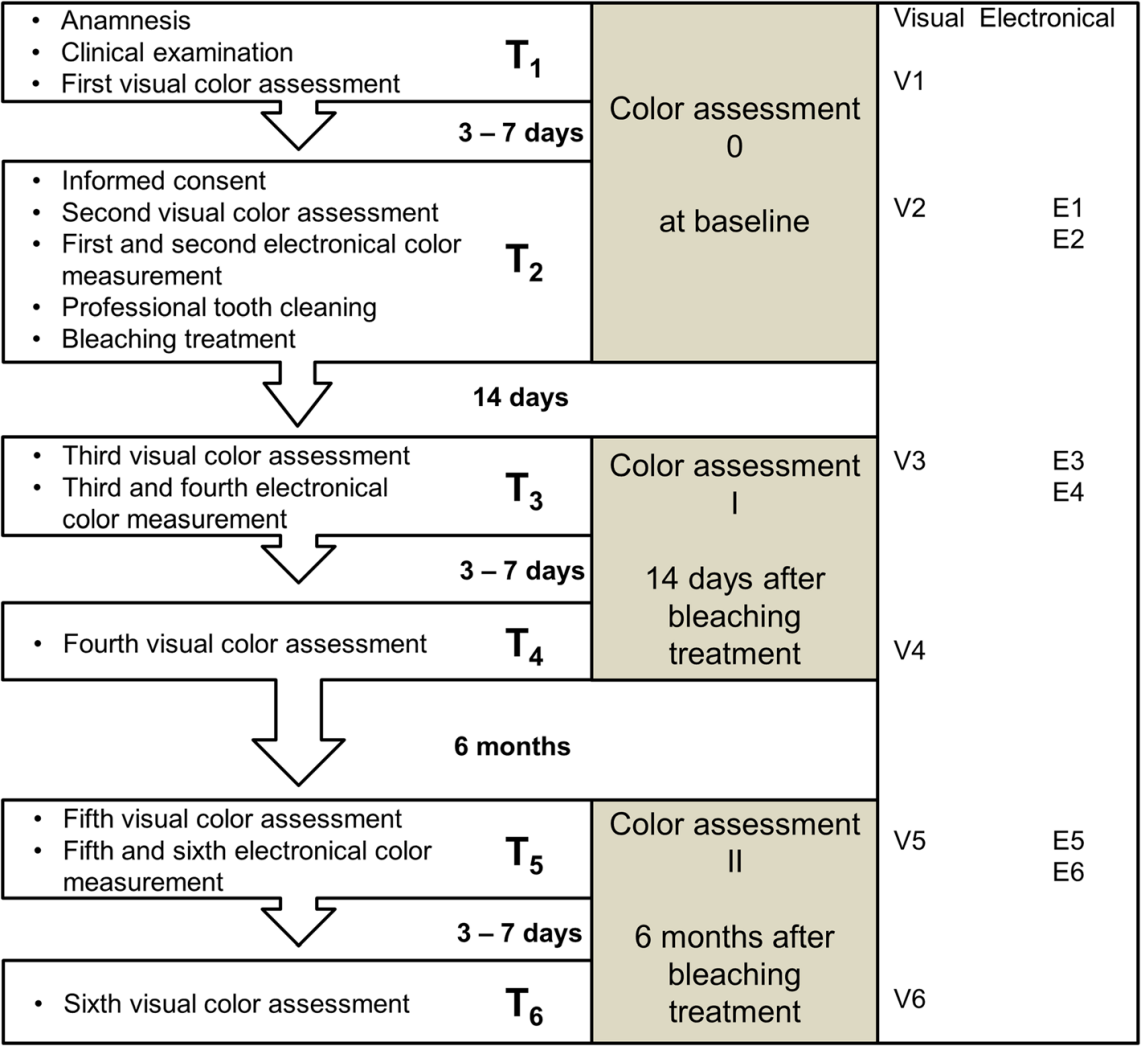
Consort Flow Diagram

The color of labial surfaces of teeth 14 to 24 was visually assessed by an experienced dental technician, who was ophthalmologically examined before this study [40], under diffuse daylight between 11 a.m. and 3 p.m. The time needed for color assessment was not restricted. Electronic measurements were performed with the spectrophotometer Shade Inspector™ (Schütz-Dental, Rosbach, Germany) by a dentist calibrated prior to this study [40]. The color systems *VITAPAN® Classical* (2D-VC; VITA Zahnfabrik, Bad Säckingen, Germany) and *VITA 3D-Master®* (3D; VITA Zahnfabrik, Bad Säckingen, Germany) served as reference systems. The VC color system has a two-dimensional structure that enables the description of *hue* (category A to D) and *lightness* including *chroma* (group 1 to 4) [41]. It serves as the standard shade guide for visual color assessment in dental practice. The 3D color system has a three-dimensional structure that enables the separate description of *lightness* (1 to 5 and 0 for bleaching), *chroma* (1 to 3, including half points), and *hue* (M, L, R) [42]. For the measurement procedure, each tooth was categorized into the gingival (S_1_), the body (S_2_), and the incisal (S_3_) segment. The incisal segment S_3_ was not included in the analysis due to its transparency. Measurements were carried out as described in the previous study [33]. Time points of visual and electronic measurements were before BT (T_1_/T_2_- Baseline), 14 days (T_3_/T_4_) and 6 months (T_5_/T_6_) after BT (Fig. 1).

### Statistical methods

ΔE = ((ΔL*)^2^ + (Δa*)^2^ + (Δb*)^2^)^1/2^ and ΔE_00_ [43] were calculated. ΔE_00_ is superior to ΔE, but its calculation is quite sophisticated. Irregularities in the color space are corrected as follows: 1. the differences in the individual dimensions are calculated; 2. weighting is carried out; 3. finally a term for the interaction between the chroma differences and the hue differences is added; the calculation includes 22 lines of formulae [43]. ΔE_00_ values are usually smaller than those of ΔE [21]. Here, we focused on ΔE because it is more commonly used. The Bland-Altman plot [44] is one of the most frequently cited methods in medicine. Although several adaptations have been discussed [45–49], we present only the classical plot with the mean difference and the limits of agreement for d(0M1), which is ΔE of each shade from 0M1. For method comparisons, but not for intra-rater comparisons, the regression line was added. Out of 840 paired observations, a total of 30–55 observations can be expected to be outside the limits of agreement according to M. Bland [50]. Besides the limits of agreement (difference between measurements ±1.96* standard deviation of the difference [44]), we present the agreement within 2.7 [16] and 3.7 [51] units of d(0M1) and ΔE. These agreement statistics and the difference between the pairs of observations (denoted by d_2_ – d_1_ for d(0M1), including standard deviation, are the only measurement error statistics also reported for ΔE. The standard error of measurement (SEM) is a further agreement statistic and reported in two versions [37], for which the values are very similar herein. The SDCD is defined as 1.96*√2*SEM ≈ 2.77*SEM [37]. The SDSC on the level of groups of observations or patient’s teeth is calculated according de Vet et al. 2001 [31]. In addition to agreement statistics, which are related to differences of repeated measurements, we present reliability statistics, which are related to calibration or comparability of raters or methods [34]. The fraction of the total measurement variance due to variance among teeth is estimated by three versions of the intraclass correlation coefficient (ICC) [28]. Whereas the ICC_(3,1)_ ignores systematic differences between the two methods, raters, or measurements of the same rater, the ICC_(2,1)_ includes an additional term of the variance among raters to account for the total measurement variance (denominator) [28, 37]. Thus, the greater the systematic difference between two raters, the smaller the ICC_(2,1)_ compared with the ICC_(3,1)_. The ICC is the most appropriate reliability statistic [37] and recommended besides the Bland-Altman plot [32]. To avoid confusing terminology, SEM, SDSC and ICC are presented in the terminology used in Shrout & Fleiss [28]. ICC and kappa, which are closely related [32, 52], are interpreted according to Byrt’s classification [53]. Graphics and statistical analyses were performed using Stata software, release 14.2 (Stata Corporation, College Station, TX, USA). As the American Statistical Association took a stand against Null Hypothesis Significance Testing [54, 55], we present confidence intervals as recommended [56]. Because accuracy requires a large sample size [44], we looked for at least 200 observations as recommended [57].

## Results

### Intra-rater variability

**Fig. 2:**
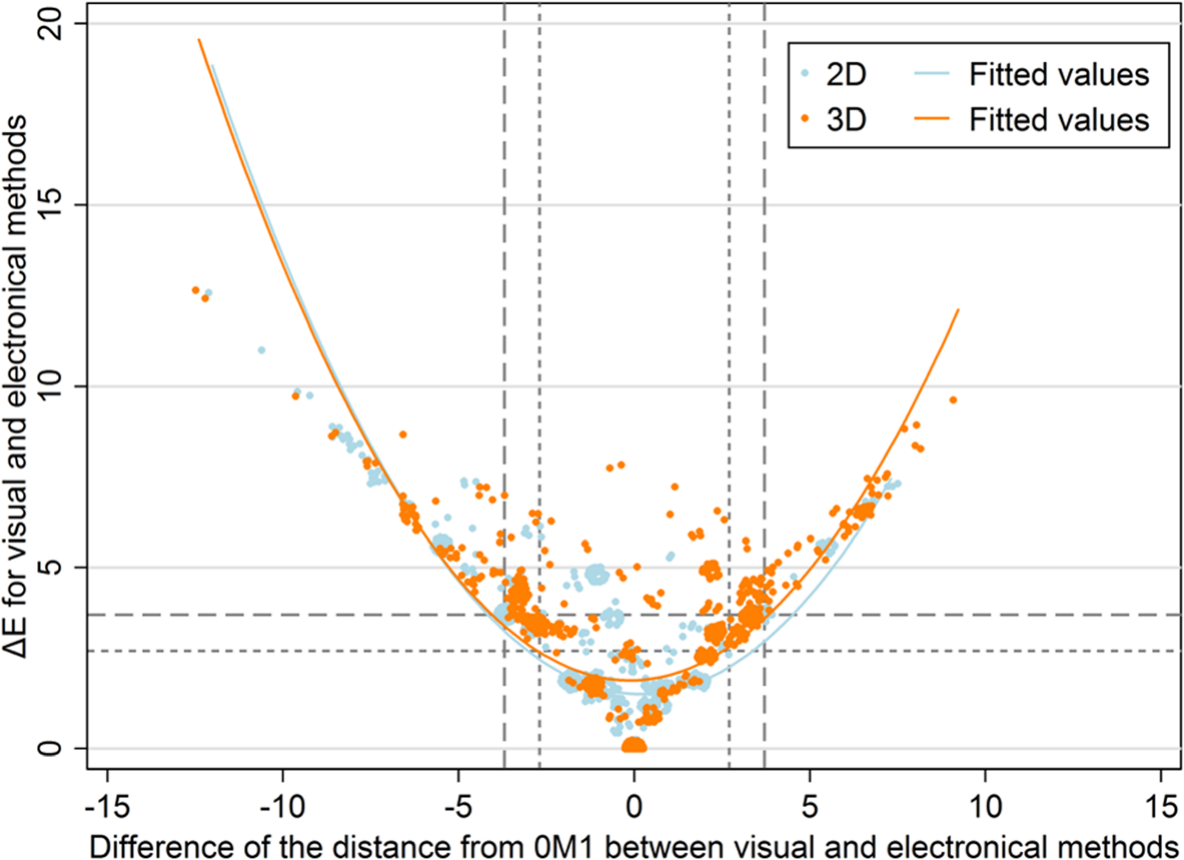
Scatter plot for the relationship between ΔE of the visual and electronic method and the difference of the distance from 0M1 between the visual and electronic method in 2D and 3D measurements; observations with the same coordinates are jittered to show their number

**Fig. 3:**
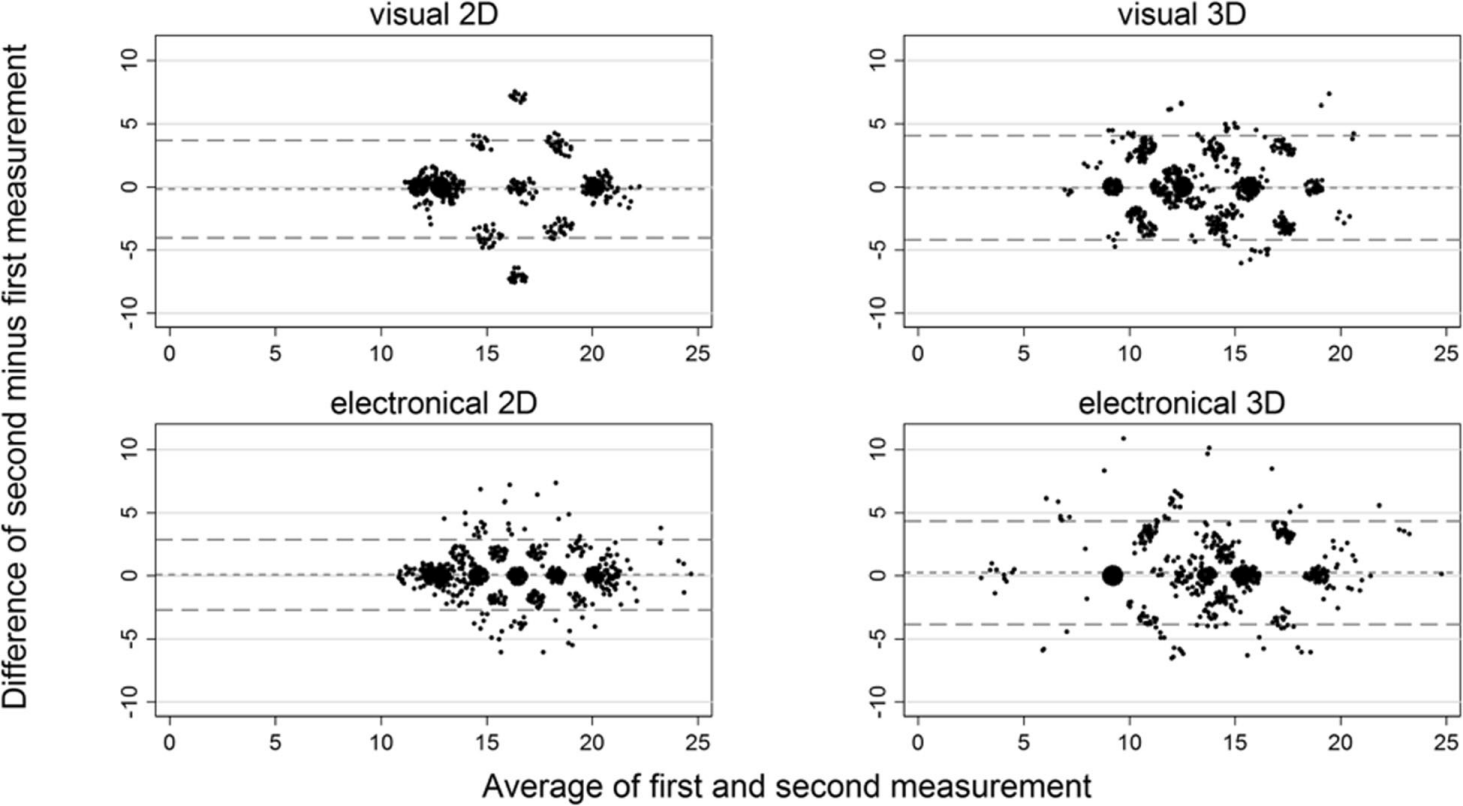
Bland-Altman plots for the distance from 0M1 (body surface); observations with the same coordinates are jittered to show their number

The agreement within the limits of ΔE < 2.7 was better for 2D than for 3D, both visually and electronically (Table 1). Figure 2 shows how the difference between two values of d(0M1) is related to ΔE, for which the difference between visual and electronical measurements was chosen. This difference in d(0M1) was strongly and substantially symmetrically related to ΔE (Fig. 2; R^2^ = 0.69 for 2D and R^2^ = 0.59 for 3D). The agreement within the limits of d(0M1| < 2.7 was also better for 2D than for 3D, both visually and electronically (Table 2). The limits of agreement were narrower for 2D_elec_ than for the remaining three methods (Table 2; Fig. 3). The Bland-Altman plots show clear patterns of disagreement for all methods, which is most pronounced for 2D_vis_ (Fig. 3). The d(0M1) range is narrowest for 2D_vis_ (11.0) and widest for 3D_elec_ (21.6) (Fig. 3); the variability of d(0M1) in terms of the pooled standard deviation is highest for 3D_elec_. The reliability in terms of the ICC is good to very good for d(0M1) (Table 2).

**Table 1:** Agreement of repeated measurements for four methods in terms of ΔE and ΔE_00_ related to a single tooth

	Visual 2D	Visual 3D	Electronical 2D	Electronical 3D
	Value	Value	Value	Value
Paired observations, number	840^a^	840 ^a^	839 ^b^	840^b^
Mean ΔE (standard deviation)	1.12 (1.95)	1.99 (1.95)	0.97 (1.41)	1.55 (2.11)
Agreement within ΔE < 2.7, proportion (95% CI)	80.1 (77.3–82.8)^**,***^	59.4 (56.0–62.7)^*,**^	90.9 (88.8–92.8)^***,†^	71.7 (68.5–74.7)^**^
Agreement within ΔE < 3.7, proportion (95% CI)	84.6 (82.0–87.0)^***^	77.9 (74.9–80.6)^**,***^	92.8 (90.9–94.5)^***,†^	83.3 (80.6–85.8)^***^
Mean ΔE_00_ (standard deviation)	0.92 (1.60)	1.59 (1.58)	0.80 (1.19)	1.27 (1.74)
Agreement within ΔE_00_ < 2.7, proportion (95% CI)	84.2 (81.5–86.6)^***^	69.5 (66.3–72.6)^**^	92.1 (90.1–93.9)^***,†^	77.4 (74.4–80.2)^**,***^
Agreement within ΔE_00_ < 3.7, proportion (95% CI)	91.9 (89.8–93.7)^***,†^	88.1 (85.7–90.2)^***^	96.3 (94.8–97.5)^†^	86.4 (83.9–88.7)^***^

^a^ V1 versus V2, V3 versus V4, V5 versus V6 acc. to the flow chart

^b^ E1 versus E2, E3 versus E4, E5 versus E6 acc. to the flow chart

Classifications for the interpretation of agreement

^*^ fair [40–60); ^**^ good [60–80); ^***^ very good [80–92); ^†^ excellent [92–100]

**Table 2:** Agreement and reliability of repeated measurements for four methods in terms of the distance from 0M1 related to a single tooth

	Visual 2D	Visual 3D	Electronical 2D	Electronical 3D
	Value	Value	Value	Value
Number of paired observations	840^a^	840^a^	839^b^	840^b^
Mean distance (SD) d_1_ from 0M1 for the 1st measurement	15.0 (3.28)	13.4 (2.89)	15.8 (2.97)	13.1 (3.69)
Mean distance (SD) d_2_ from 0M1 for the 2nd measurement	14.9 (3.23)	13.3 (2.76)	15.9 (2.94)	13.4 (3.73)
Pooled SD of the 1st and 2nd measurement	3.25	2.83	2.96	3.71
Difference d_2_ – d_1_ (standard deviation)	−0.17 (1.98)	−0.08 (2.11)	0.09 (1.42)	0.26 (2.09)
Agreement within |d(0M1)| < 2.7, proportion (95% CI)	83.7 (81.0–86.1)^***^	70.6 (67.4–73.7)^**^	93.6 (91.7–95.1)^***,†^	77.3 (74.3–80.1)^**,***^
Agreement within |d(0M1)| < 3.7, proportion (95% CI)	94.0 (92.2–95.6)^†^	94.0 (92.2–95.6)^†^	97.0 (95.6–98.1)^†^	93.1 (91.2–94.7)^***,†^
Limits of agreement	−4.04 – 3.70	− 4.21 – 4.06	−2.70 – 2.88	−3.84 – 4.36
Number of observations outside the limits of agreement total (lower; higher); expected: 30–55	50 (38; 12)	38 (13; 25)	53 (26; 27)	52 (20; 32)
Largest mean d(0M1) value	22.2	20.7	24.8	24.9
Smallest mean d(0M1) value	11.2	7.3	11.2	3.3
SEM_(2,1)_	1.400	1.489	1.007	1.489
SEM_(3,1)_	1.396	1.489	1.005	1.479
SDCD_(2,1)_	3.88	4.13	2.79	4.13
SDCD_(3,1)_	3.87	4.13	2.79	4.10
ICC_(1,1)_ (95% CI)	0.81 (0.79–0.84)^**,***^	0.72 (0.69–0.75)^**^	0.88 (0.87–0.90)^***^	0.84 (0.82–0.86)^***^
ICC_(2,1)_ (95% CI)	0.81 (0.79–0.84)^**,***^	0.72 (0.69–0.75)^**^	0.88 (0.87–0.90)^***^	0.84 (0.82–0.86)^***^
ICC_(3,1)_ (95% CI)	0.82 (0.79–0.84)^**,***^	0.72 (0.69–0.75)^**^	0.88 (0.87–0.90)^***^	0.84 (0.82–0.86)^***^

*SD* denotes standard deviation, *CI* denotes confidence interval, *SEM* denotes standard error of measurement, *SDCD* denotes smallest detectable color difference, *ICC* denotes intraclass correlation coefficient

^a^ V1 versus V2, V3 versus V4, V5 versus V6 acc. to the flow chart

^b^ E1 versus E2, E3 versus E4, E5 versus E6 acc. to the flow chart

Classifications for the interpretation of agreement

^**^ good [60–80); ^***^ very good [80–92); ^†^ excellent [92–100]

Classifications for the interpretation of reliability in terms of ICC

^*^ fair [0.4–0.6); ^**^ good [0.6–0.8); ^***^ very good [0.8–0.92); ^†^ excellent [0.92–1.0]

As hypothesized for the three single dimensions, *hue* is less reliable than *lightness* or *chroma,* both electronically (Kappa value for *hue* = 0.45, 95% CI: 0.40–0.50; ICC_(1,1)_ for *lightness* = 0.76, 95% CI: 0.74–0.79; ICC_(1,1)_ for *chroma* = 0.67, 95% CI: 0.63–0.70) and visually (Kappa value for *hue* = 0.01, 95% CI: − 0.05 – 0.06; ICC_(1,1)_ for *lightness* = 0.52, 95% CI: 0.47–0.57; ICC_(1,1)_ for *chroma* = 0.66, 95% CI: 0.62–0.69).

The standard errors of measurement and SDCDs were essentially the same for the four methods, except for 2D_elec_, which was better (Table 2). On the level of groups of observations or patient’s teeth, the SDCD of 2D_elec_ diminished from 2.8 for a single tooth to 1.4 and 1.0 for four and eight teeth, respectively. The SDCD of 2D_vis_ decreased from 3.9 for a single tooth to 1.9 and 1.4 for four and eight teeth, respectively.

### Inter-method variability

The comparability of visual and electronic measurements was fair to good in 2D and slight to fair in 3D for the agreement within the limits of ΔE < 2.7 (Table 3). The corresponding agreement of 2D and 3D measurements was fair in the visual approach, and poor to slight in the electronic approach (Table 3).

**Table 3:** Comparing methods of measurements in terms of ΔE and ΔE_00_: 2D versus 3D within visual or electronical measurement; visual versus electronical measurements within 2D and 3D

	Visual versus electronical		2D versus 3D	
	within 2D	within 3D	within visual	within electronical
	Value	Value	Value	Value
Paired observations, number	839^a^	840^a^	1680^b^	1679^c^
Mean ΔE (standard deviation)	2.53 (2.17)	2.99 (2.21)	3.46 (1.66)	3.91 (1.29)
Agreement within ΔE < 2.7, proportion (95% CI)	59.6 (56.2–62.9)^*,**^	40.6 (37.3–44.0)^-,*^	45.2 (42.8–47.6)^*^	18.6 (16.7–20.5)^--,-^
Agreement within ΔE < 3.7, proportion (95% CI)	67.2 (63.9–70.4)^**^	68.5 (65.3–71.7)^**^	52.9 (50.5–55.3)^*^	46.6 (44.2–49.0)^*^
Mean ΔE_00_ (standard deviation)	2.08 (1.80)	2.37 (1.82)	3.26 (1.23)	3.50 (1.00)
Agreement within ΔE_00_ < 2.7, proportion (95% CI)	62.9 (59.6–65.5)^*,**^	56.0 (52.5–59.3)^*^	45.8 (43.4–48.2)^*^	23.5 (21.5–25.6)^−^
Agreement within ΔE_00_ < 3.7, proportion (95% CI)	82.1 (79.4–84.7)^**,***^	75.2 (72.2–78.1)^**^	71.7 (69.5–73.9)^**^	64.6 (62.3–66.9)^**^

^a^ V2 versus E1, V3 versus E3, V5 versus E5 acc. to the flow chart

^b^ D2 versus D3 measurements for V1 – V6 acc. to the flow chart

^c^ D2 versus D3 measurements for E1 – E6 acc. to the flow chart

Classifications for the interpretation of agreement

^−−^ poor < 20; ^−^ slight [20–40); ^*^ fair [40–60); ^**^ good [60–80); ^***^ very good [80–92)

The comparability of visual and electronic measurements was good in 2D and fair in 3D for the agreement within the limits of |d(0M1)| < 2.7 (Table 4). The corresponding agreement of 2D and 3D measurements was good in the visual approach, and fair in the electronic approach (Table 4).

**Table 4:** Comparing methods of measurements of the distance from 0M1 related to a single tooth: 2D versus 3D within visual or electronical measurement; visual versus electronical measurements within 2D and 3D

	Visual versus electronical		2D versus 3D	
	within 2D	within 3D	within visual	within electronical
	Value	Value	Value	Value
Number of paired observations	839^a^	840^a^	1680^b^	1679^c^
Mean distance (SD) d_1_ from 0M1 for the electronical measurement	15.8 (2.97)	13.1 (3.69)		
Mean distance (SD) d_2_ from 0M1 for the visual measurement	14.9 (3.28)	13.4 (2.88)		
Mean distance (SD) d_1_ from 0M1 for the 2D measurement			15.0 (3.25)	15.9 (2.96)
Mean distance (SD) d_2_ from 0M1 for the 3D measurement			13.3 (2.82)	13.3 (3.71)
Difference d_2_ – d_1_ (standard deviation)	−0.89 (2.77)	0.22 (3.05)	−1.64 (1.98)	−2.58 (1.70)
Agreement within |d(0M1)| < 2.7, proportion (95% CI)	69.1 (65.9–72.2)^**^	53.3 (49.9–56.7)^*^	66.5 (64.2–68.8)^**^	47.1 (44.6–49.5)^*^
Agreement within |d(0M1)| < 3.7, proportion (95% CI)	86.3 (83.8–88.5)^***^	86.3 (83.8–88.6)^***^	80.9 (78.9–82.7)^**,***^	84.0 (82.2–85.8)^***^
Limits of agreement	−6.33 – 4.55	−5.76 – 6.19	− 5.53 – 2.25	− 5.90 – 0.75
Number of observations outside the limits of agreement total (lower; higher)	58^d^ (33; 25)	60^d^ (30; 30)	82^e^ (21; 61)	49^e^ (34; 15)
ICC_(2,1)_ (95% CI)	0.58 (0.50–0.65)^*,**^	0.58 (0.53–0.62)^*,**^	0.69 (0.27–0.84)^-,***^	0.67 (−0.06–0.88)^--,***^
ICC_(3,1)_ (95% CI)	0.61 (0.56–0.65)^*,**^	0.58 (0.53–0.62)^*,**^	0.79 (0.77–0.81)^**,***^	0.87 (0.86–0.88)^***^

^a^ V2 versus E1, V3 versus E3, V5 versus E5 acc. to the flow chart

^b^ D2 versus D3 measurements for V1 – V6 acc. to the flow chart

^c^ D2 versus D3 measurements for E1 – E6 acc. to the flow chart

^d^ expected number: 30–55

^e^ expected number: 66–102

Classifications for the interpretation of agreement

^−−^ poor < 20; ^−^ slight [20–40); ^*^ fair [40–60); ^**^ good [60–80); ^***^ very good [80–92)

Classifications for the interpretation of reliability in terms of ICC

^−−^ poor < 0.2; ^−^ slight [0.2–0.4); ^*^ fair [0.4–0.6); ^**^ good [0.6–0.8); ^***^ very good [0.8–0.92)

**Fig. 4:**
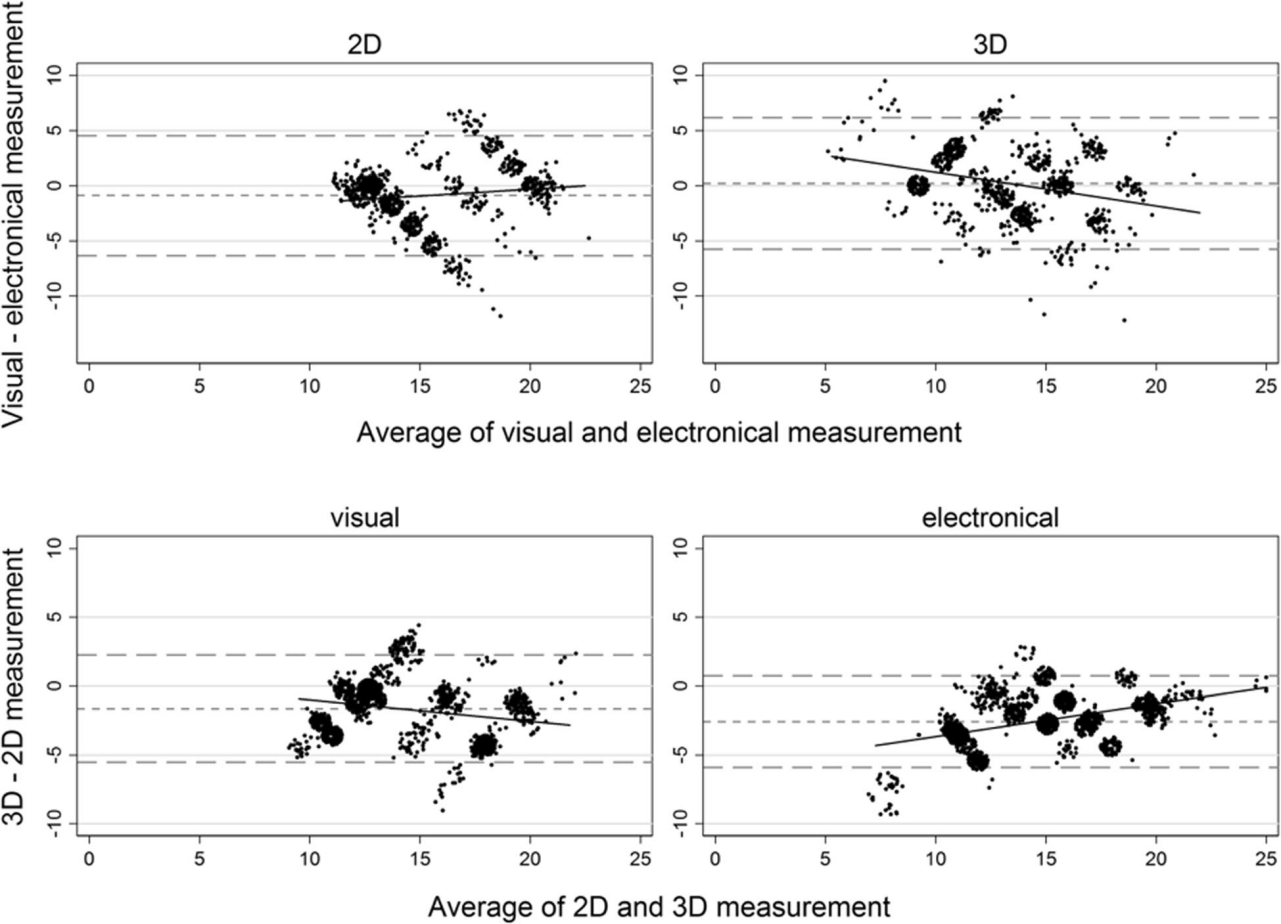
Bland-Altman plots for the distance from 0M1 (body surface); observations with the same coordinates are jittered to show their number

Concerning the comparability of the visual and electronic measurements, the difference d_2_ – d_1_, which indicates systematic error, was moderate in 2D and small in 3D (Table 4; Fig. 4). The Bland-Altman plots show marked patterns of disagreement for the approaches.

Concerning the comparability of 2D and 3D measurements, the difference d_2_ – d_1_ indicates systematic error, which was pronounced in the electronic approach (Table 4; Fig. 4). This difference can be interpreted as constant bias. Assuming proportional bias, the regression line can be cautiously interpreted. The Bland-Altman plots, however, showed clear patterns of disagreement for the approaches; the bias between the 2D and 3D system is neither constant nor uniquely proportional.

The reliability in terms of the ICC was fair to good for visual and electronic measurements. The reliability in terms of the ICC_(3,1)_, which ignores systematic differences, was good to very good for 2D and 3D measurements. The reliability in terms of the ICC_(2,1)_, which takes into account systematic differences, was poor to very good.

## Discussion

The 2D system proved superior to the 3D system both visually and electronically in terms of ΔE and d(0M1) for statistics of agreement and reliability to assess intra-rater variability. All four methods showed strong patterns of disagreement between repeated measurements in Bland-Altman plots. As hypothesized, the 3D system is less reliable for *hue* than for *lightness* and *chroma*, a phenomenon which was more pronounced visually than electronically. The SDCD differs by the four methods used and was most favorable in the electronic 2D system. The agreement between the 2D and 3D systems in terms of ΔE was not *good*. It was lower in the electronic than in the visual method. The comparability of the 2D and 3D systems was uncertain, because confidence intervals of ICCs accounting for systematic error were wide. The systematic error between the 2D and 3D systems cannot be neglected. The reliability of the visual and electronic method was substantially the same in the 2D and 3D systems; this comparability was *fair* to *good.*

Below, the following aspects are discussed: 2D and 3D, visual and electronic, ΔE and d(0M1), Bland-Altman plots and statistics (patterns and numbers), single shade designations of the 3D system, validity and reliability, statistical SDCD and known thresholds, agreement and reliability (comparability), human and machine, and intra- and inter-method variability.

### 2D and 3D systems

The 2D and 3D systems differ in the color space assessed [33]. Some 3D shades that are lighter (*lightness*) or stronger (*chroma*) are not well covered by the 2D system, which is especially pronounced for the additional bleaching shades available only in the 3D system. Compared to VC, *hue* ranges of 3D Master are extended toward yellow-red, and 3D Master shades are more uniformly spaced than that of VC [6]. In contrast, there are spatial gaps in the 3D system which are filled in the 2D system [33, 41]. In short, both guides are suboptimal and can be improved [14].

The variability between raters may favor the 3D Master shade guide over the VC shade guide [58]. The coverage error favors the 3D system, although it is unclear whether the difference between the 2D and 3D systems is clinically relevant [12, 14, 59–61]. However, the clear patterns in Bland-Altman plots for d(0M1) cast doubt on the meaningfulness of converting 3D shades into VC shades (2D) as suggested elsewhere [62].

### Visual and electronical method

The gaps mentioned above that are filled by the 2D system are supported by additional 2D shades to assess quarter-points for the second shade designation number [33], which is an important difference between the visual and electronic method. A further important difference is the extension of the second shade designation number from the visual four-point scale to the electronic five-point scale. Similarly, the electronic 3D system includes bleaching shades not used by the visual 3D system evaluated here. Thus, it could have been expected that a human rater is inferior to the electronic rater, especially for the 2D system. It is of note that the agreement of intra-rater variability in terms of ΔE and d(0M1) is better for the visual 2D measurement than that for the electronic 3D measurement.

Several studies have found that instrumental methods are more accurate or reliable than visual measurements [11, 19, 23–25, 63–65]. A recent study, however, has shown that clinically relevant differences between the visual evaluation and the intraoral scanning device (3Shape) are negligible [20]. According to Li & Wang, the reliability of shade matching can be ensured neither by the instrumental nor by the visual approach [66]. Furthermore, the difference in color matching between human-eye assessment and computerized colorimetry depends on tooth type [18] and shade [8].

### ΔE and d(0M1)

ΔE supports only statistics on agreement; neither Bland-Altman plots nor reliability statistics are feasible. Essentially, d(0M1) enables evaluating patterns of disagreement, other agreement statistics such as SDCD, and reliability statistics including versions of ICC accounting for systematic errors. Regarding agreement of repeated measurements of the same rater, the differences among the four methods are substantially the same for ΔE < 2.7 and d(0M1). The level of agreement within fixed limits, however, is higher for d(0M1). For example, d(0M1) hardly differentiates 3M1 from 2L2.5 (d(0M1): 15.2 and 15.3, respectively) although ΔE is 8.3. Thus, if *lightness* is compensated by less *chroma* (or *chroma* by darkness), then d(0M1) will not work well. The systematic errors between 2D and 3D measurements in d(0M1) are plausible, because the 2D and 3D systems differ in the color space assessed (see above). Within the 2D system, systematic errors between visual and electronic measurements are small, which can be explained by the additional quarter-point shades in the electronic 2D system.

### Bland-Altman plots and statistics – patterns and numbers

According to Bland-Altman plots, bias between the 2D and 3D systems is neither constant nor uniquely proportional. Even if these kinds of bias could be adjusted for **–** as suggested for uniquely proportional bias [48, 49] **–** the clear patterns are not appropriate for sophisticated statistical methods. Thus, Bland-Altman plots provide important information hardly available in numbers.

### Single shade designations of the 3D system and d(0M1)

Although the reliability for the *hue* component of the visual 3D system is zero, the corresponding d(0M1) indicates good reliability. Likewise, the reliabilities are *fair* versus *very good* for the electronical 3D system, respectively. Thus, reliabilities of single shade designations can be misleading, especially for *hue*, for which ΔE values are only about 1.5 (see above). Nevertheless, the *hue* component of the 3D system is problematic, because its reliability is lower than those of *lightness* and *chroma*.

### Validity and reliability

Colorimetry does not facilitate valid measurements. The value of d(0M1), however, supports pseudo-valid measurements, as the range of d(0M1) values differs across the four methods. The bleaching shades added to the electronic 3D system (not to the visual 3D system) make the difference: this range (21.6) is twice as high compared to visual 2D (11.0). Reliability in terms of the ICC depends on this range – if the variability of d(0M1) is small, the ICC will be small. As expected, the pooled standard deviation of the electronic 3D system is higher than that of the electronic 2D system. The ICC of the electronic 3D system, however, is lower, which emphasizes the problems with the 3D system – independent of human raters.

### Smallest detectable color difference, acceptable and perceptible thresholds

An acceptability threshold of 2.7 in ΔE and a perceptibility threshold of 1.2 in ΔE are known [16]. The SDCD in terms of d(0M1) depends on the method and decreases from 2.8 to 1.0 for a row of eight teeth using electronic 2D measurements. These are statistical values and can differ from study to study. However, it is plausible that electronic 2D is the method with the best agreement, including SDCD. For properties of ΔE and d(0M1), electronic 2D is the recommended method for study designs with repeated measurements, such as longitudinal studies.

### Agreement and reliability (comparability)

Whereas intra-rater agreement of repeated measurements in terms of SEM and SDCD does not differ between visual and electronic 3D measurements, the reliability or ICC differ substantially. Thus, a single human rater is not worse than the electronic device for a longitudinal study when using the 3D system. The comparability of the four methods remains uncertain. Therefore, the same method should also be used in multicenter studies.

### Human and machine

Compared with a set of human raters, a set of devices from the same electronic system should have higher levels of standardization [67], which corresponds to the more favorable ICCs observed. However, n-of-1 trials, as used herein for the single human rater, limit generalizability. It may be further argued that the human rater lacks the ability to perceive *hue* [39]. But even if the examiner had lacked this ability, this would not have invalidated our conclusions, because we did not make an isolated statement on *hue,* but rather compared *hue* with *lightness* and *chroma*. These intra-human comparisons are supported by the n-of-1 trial design. Moreover, the same intra-device comparisons support the hypothesis that *hue* is not well reproducible; the electronic reliability of *hue* is merely *fair*. In addition to our findings, background knowledge further supports that 3D *hue* cannot be well assessed (see Introduction).

### Intra- and inter-method variability – validity revisited

Whereas the reliability within each of the four methods is *good* to *very good*, comparability of the visual and electronic measurements is only *fair* to *good*. This also questions the validity of visual and electronic measurements. In turn, this question also refers to the difference between the 2D and 3D system. In fact, Bland-Altman plots using the 2D system suggest that both visual and electronic values are valid only for d(0M1) values of about 12 (A1 – A2, B1 – B2) and greater than 20 (A4, B3 – B4, C3 – C4, D4). The shades B1 and A2 are not well covered by the 3D system [33], which is mirrored in the corresponding Bland-Altman plots. Vice versa, 3D shades 1M1 and 1M2 (both d(0M1)<11.2 for the minimum of the 2D system) are not well covered by the 2D system [33] and question the validity of adjacent 2D shades, namely A1, B1, and B2. In daily practice, the 3D system may be useful for shades not available in the 2D system. Nevertheless, switching between methods cannot be recommended in scientific studies. The 3D system, however, can be favorable in bleaching studies owing to the added bleaching shades.

## Conclusion

The 3D system may confuse both human raters and electronic devices. The 2D system is the simple and best choice.

## Data Availability

All data are available on request at the Department of Orthodontics, Dental School, University Medicine, Walther-Rathenau-Strasse 42, 17475 Greifswald, Germany.

## Abbreviations

2D_vis_: 2D_visual_
2D_elec_: 2D_electronical_
3D_vis_: 3D_visual_
3D_elec_: 3D_electronical_
